# Age, Not Sex, Drives Sarcopenia Severity in Mexican Older Adults with a Health Insurance Plan

**DOI:** 10.3390/geriatrics11040077

**Published:** 2026-07-01

**Authors:** Selma Karime Castillo-Vazquez, Nadia Alejandra Rivero-Segura, Juan Carlos Gomez-Verjan, Jazmin Camacho, Daniel Hernández-Pando

**Affiliations:** 1 Dirección de Investigación, Instituto Nacional de Geriatría, Ciudad de México 10200, Mexico; selmakarime@gmail.com (S.K.C.-V.); nrivero@inger.gob.mx (N.A.R.-S.); jverjan@inger.gob.mx (J.C.G.-V.); 2 Dirección de Salud Poblacional, Koltin, Ciudad de México 06700, Mexico; jazmin@koltin.com.mx

**Keywords:** sarcopenia, aging, sex differences, muscle mass, muscle strength

## Abstract

**Background/Objectives**: Sarcopenia significantly increases the risk of hospitalization and mortality in older adults. However, the prevalence by sex varies across populations and according to the criteria used by the European Working Group on Sarcopenia in Older People 2 (EWGSOP2). Hence, in the current study, we aim to characterize the prevalence of sarcopenia by jointly evaluating all three diagnostic domains in a Mexican cohort of older adults subscribed to a health insurance plan. **Methods**: We performed binomial and multinomial regression models, ordinal logistic regression, and multiple linear regression from the data corresponding to muscle mass (ASMI), muscle strength (handgrip dynamometry), and physical performance (SPPB) from 556 Mexican older adults (62.2% female; 72.27 ± 6.35 y.o.) categorized by sarcopenia severity according to the EWGSOP2. **Results**: Men exhibited significantly higher absolute muscle mass and strength across all categories (*p* < 0.001). Additionally, our statistical analyses demonstrate that age, but not sex, is involved in sarcopenia severity in this population. Moreover, when analyzing the disaggregated EWGSOP2 domains, the results demonstrate that sex is significantly associated with muscle mass (ASMI) and muscle strength (Handgrip strength). **Conclusions**: These results suggest that sex only influences both muscle mass (ASMI) and muscle strength (handgrip strength) while sarcopenia severity only depends on age.

## 1. Introduction

Currently, the major determinant of frailty, functional dependence, and increased risk of hospitalization and mortality in geriatric populations, according to [1], is sarcopenia, defined as the progressive and generalized loss of skeletal muscle mass, strength, and physical performance [2]. To assess muscle performance, diagnostic frameworks such as the European Working Group on Sarcopenia in Older People 2 (EWGSOP2) and Asian Working Group for Sarcopenia (AWGS) 2019 consensus criteria now recognize three interrelated domains: muscle mass (typically assessed through imaging techniques such as Dual-Energy X-ray Absorptiometry (DEXA)), muscle strength (most commonly via handgrip dynamometry), and physical performance (e.g., gait speed or chair-stand tests). Impairment in three domains generally makes the condition more severe and associated with a higher likelihood of adverse outcomes. Global prevalence of sarcopenia is approximately 10% among older adults [3]; however, other studies reported that sarcopenia prevalence is higher in women [1]. This higher prevalence in women may be explained by the accelerated muscle loss following estrogenic decline after menopause, lower baseline muscle mass compared to men, and greater relative decreases in muscle strength with aging [4].

This effect has been attributed to methodological issues such as the use of sex-specific cutoff points derived from reference populations that may not adequately reflect muscle loss in women, as well as variability across diagnostic frameworks (e.g., EWGSOP2, AWGS, and Foundation for the National Institutes of Health framework) in the thresholds applied for muscle mass, strength, and physical performance [5]. Indeed, reported sarcopenia prevalence varies widely depending on the diagnostic criteria used, with ranges of 0.3–91.2% in women and 0.4–87.7% in men across studies [3,6], suggesting that current tools may not consistently capture sex-related differences in muscle decline. These inconsistencies in sex-related differences in sarcopenia diagnosis highlight the need for more sex-sensitive and population-specific diagnostic criteria [7].

On the other hand, to overcome such limitations, several studies conducted comprehensive evaluations that included DEXA to estimate muscle mass, handgrip dynamometry to assess muscle strength, and, in some cases, physical performance tests. Interestingly, the results demonstrate that the prevalence of sarcopenia varies widely depending on the diagnostic criteria used [8,9,10]. Moreover, these data suggest that evaluating these parameters altogether may improve the diagnosis of sarcopenia, leading to improved medical attention and preventing adverse outcomes. For instance, data obtained from the FraDySMex cohort, integrating DEXA-derived muscle mass, handgrip strength, and additional functional indicators, suggest that women may exhibit greater impairment, particularly in mass and function, consistent with patterns previously described [11]. This pattern is not exclusive to the Mexican population. In a landmark US study, the prevalence of class I and class II sarcopenia was greater in older women than in older men, and the likelihood of functional impairment and disability was approximately three times greater in women with severe sarcopenia compared to only two times greater in men [12]. Similarly, a Japanese community-based cohort found that approximately one-half of women aged 80 years and older had sarcopenia, compared to one-third of men [13]. In Korea, the prevalence of sarcopenia among adults aged 65–74 years was 26.4% in females versus 19.2% in males [14]. Furthermore, the decline in lower limb muscle strength and balance ability has been shown to be greater in older women than in older men [14,15], further supporting the notion that functional impairment in sarcopenia follows a sex-specific pattern that transcends geographic and ethnic boundaries.

Nevertheless, since current evidence about sex differences in sarcopenia prevalence remains limited and fragmented, it is difficult to determine whether the severity or prevalence of sarcopenia is consistently higher in other populations. Hence, in the current study, we aim to characterize the prevalence of sarcopenia by jointly evaluating all three diagnostic domains in a Mexican cohort of older adults. Since in the Mexican context—characterized by rapid population aging and a high burden of chronic comorbidities—the lack of studies analyzing sex-stratified severity constrains the development of targeted screening strategies, prioritization of interventions, and personalization of rehabilitation and nutritional therapies. Understanding whether severity differs by sex is clinically relevant because: (a) it would determine whether men and women require different follow-up criteria or intervention intensities; (b) it would help anticipate sex-specific risks of falls, dependence, and cognitive decline; and (c) it would guide public health policies and geriatric rehabilitation resources tailored to the local demographic reality.

## 2. Materials and Methods

### 2.1. Study Design and Population

A cross-sectional study was conducted using data from the Longevity Study, derived from records of beneficiaries of Koltin, a private health insurance plan for older adults in Mexico (https://koltin.mx/membresia/seguro-de-gastos-medicos-mayores accessed on 15 July 2026). This study includes a comprehensive assessment of body composition, muscle strength, and physical performance to identify conditions associated with biological and functional aging. For the present analysis, we used the clinical and functional records obtained during evaluations performed from January 2025 to October 2025.

We included older adults who completed all the following assessments: dual-energy X-ray absorptiometry (DEXA) to evaluate muscle mass, handgrip dynamometry, and the Short Physical Performance Battery (SPPB).

### 2.2. Clinical and Functional Assessments

#### 2.2.1. Body Composition via DEXA

Appendicular skeletal muscle mass (ASM) was measured using a Horizon DEXA densitometer (Hologic Horizon Wi). The appendicular skeletal muscle mass index (ASMI) was calculated by dividing ASM by height squared (kg/m^2^). All scans were conducted by certified personnel, and equipment calibration followed international standards.

#### 2.2.2. Muscle Strength (Handgrip Dynamometry)

Handgrip strength was assessed using a hydraulic dynamometer (Baseline Deluxe), following the guidelines of the American Society of Hand Therapists [16]. Three measurements were collected by hand, and the highest value was used for analysis. Participants performed the test in a seated position, with the shoulder adducted, elbow flexed at 90°, and forearm in a neutral position.

#### 2.2.3. Physical Performance

Physical performance was evaluated using the Short Physical Performance Battery (SPPB), which includes static balance, 4 m gait speed, and the five-time chair-stand test [17,18]. Scores range from 0 to 12, with lower values indicating poorer functional performance. Although population-specific normative values for the SPPB in Mexican community-dwelling older adults are not yet available, the Spanish-language version of the SPPB has been previously validated and used in Mexican and Mexican-American populations [19], and its scoring criteria are internationally standardized, supporting its use as a cross-culturally comparable measure of physical performance.

### 2.3. Definition and Classification of Sarcopenia

Sarcopenia was defined according to the criteria of the European Working Group on Sarcopenia in Older People 2 (EWGSOP2), as previously reported among older adults in Mexico City [20]. Three levels of severity were established in mild, moderate, and severe sarcopenia based on the combination of reduced muscle strength (handgrip dynamometry female < 16 kg and male < 27 kg), low muscle mass (ASMI female < 5.49 kg/m^2^ and male < 6.99 kg/m^2^), and impaired physical performance (SPPB score ≤ 8), according to the proposal for EWGSOP2. Considering mild sarcopenia is defined as low muscle mass, moderate sarcopenia is defined as low muscle mass with reduced muscle strength or impaired physical performance. Severe sarcopenia involves a reduction in all three criteria (low muscle mass, reduced muscle strength, and impaired physical performance).

### 2.4. Statistical Analysis

Sex-related differences in sarcopenia severity were analyzed using a structured, multimodal statistical approach. Sarcopenia severity was originally classified according to EWGSOP2 criteria into three categories (mild, moderate, severe). The moderate and severe categories were combined into a single clinically relevant group (“moderate-to-severe sarcopenia”) for specific analyses. The multimorbidity variable was constructed based on considering more than two chronic diseases. Gender differences in the domains of muscle mass (ASMI), handgrip strength (DINA), and physical performance (SPPB score) were analyzed using nonparametric Mann–Whitney tests in GraphPad Prism^®^ v8.4.2. Multiple linear regressions were performed to identify the factors associated with higher or lower ASMI, handgrip strength, and physical performance (SPPB). ASMI, handgrip strength, or SPPB score were used as dependent variables, while age, BMI, sex, and multimorbidity were considered independent variables. To examine whether sex independently predicted clinically significant sarcopenia severity, a binary logistic regression model was constructed using the grouped dichotomous outcome (mild vs. moderate-to-severe sarcopenia). Sex (female as the reference category) served as the primary predictor. Odds ratios with 95% confidence intervals were estimated, and model-based predicted probabilities with confidence intervals were graphically displayed to provide an interpretable representation of clinical risk. To preserve the full ordinal information of the original EWGSOP2 severity scale, a multinomial logistic regression model and ordinal logistic regression were additionally fitted to the three uncollapsed severity levels. This allowed estimation of sex-specific predicted probabilities for mild, moderate, and severe sarcopenia. The models were adjusted for age, sex, BMI, and multimorbidity. All models were fitted using RStudio (version 4.5.1) with the tidyverse (version 2.0.0), broom (version 1.0.13), ggplot2 (version 4.0.3), nnet (version 7.3–20), MASS (7.3–65), and car (3.1–5) packages. A two-sided significance level of *p* < 0.05 was applied throughout.

### 2.5. Ethical Statement

This analysis relied exclusively on secondary data obtained from the electronic health system of the Koltin Longevity Study. All participants provided written informed consent when required by the original study protocol. Additionally, this study was approved by Comités de Investigación, Ética en Investigación y Bioseguridad at the Instituto Nacional de Geriatría with the number DI-PI-003/2026. Data protection and privacy measures complied with applicable Mexican laws and guidelines. Clinical data were anonymized by assigning a unique identification code to each participant to protect identity and avoid duplication in subsequent analyses. All methods employed in the study adhered to the Declaration of Helsinki and the Ley General de Salud of Mexico.

## 3. Results

Our sample consisted of 556 participants (female, *n* = 346; male, *n* = 210), with an overall mean age of 72.27 ± 6.35 (Table 1). Mean age was 71.49 ± 6.49 in women and 73.57 ± 5.92 y.o. in men. The minimum age recorded was 52 years in the total sample (52 in women and 54 in men), while the maximum age was 86 years overall (85 in women and 86 in men). According to our results, women showed a higher proportion of mild sarcopenia. Nevertheless, the distribution of sarcopenia severity between men and women is not statistically significant (overall chi-square test, *p*-value = 0.062).

### 3.1. Differences in Muscle Mass Measured by the ASMI Are Associated with Age, Sex, and BMI

**Figure 1:**
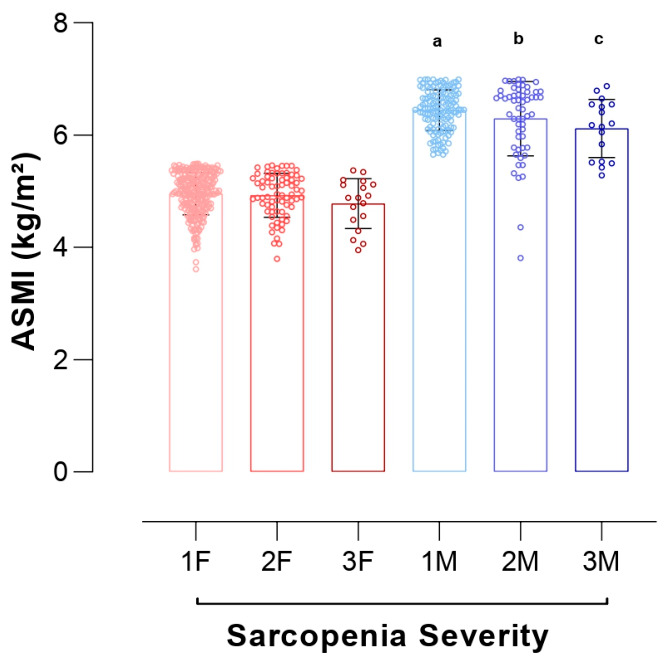
Muscle mass (kg/m^2^) is higher in men than in women across all categories of sarcopenia severity. This plot shows muscle mass measured by ASMI (kg/m^2^) in females (red) and males (blue) across all categories of sarcopenia severity. The absolute values for ASMI in each group are expressed as mean ± S.D. For females: 1F corresponds to mild sarcopenia (4.96 ± 0.38 kg/m^2^, a); 2F corresponds to moderate sarcopenia (4.93 ± 0.39 kg/m^2^, b); and 3F corresponds to severe sarcopenia (4.78 ± 0.44 kg/m^2^, c). For males: 1M corresponds to mild sarcopenia (6.44 ± 0.36 kg/m^2^); 2M corresponds to moderate sarcopenia (6.29 ± 0.66 kg/m^2^); and 3M corresponds to severe sarcopenia (6.12 ± 0.52 kg/m^2^). Lowercase letters (a, b, c) indicate significant differences according to *a:* 1F vs. 1M; *b:* 2F vs. 2M; and *c:* 3F vs. 3M (*p*-values < 0.001).

Since we observed a trend toward increasing prevalence of sarcopenia among women, we aim to explore whether, by disaggregating the three domains, this difference becomes significant. First, we analyze muscle mass by sex (males and females). As depicted in Figure 1, men had significantly higher ASMI than women across all categories of sarcopenia severity (*p*-value < 0.001). Although a slight decreasing trend in ASMI values was observed descriptively, this pattern was not statistically significant within the female subgroup. The absence of a non-sarcopenic female reference group within our cohort limits direct within-sample comparisons of muscle mass across sarcopenia status in women; future studies should include this comparison using population-specific normative data.

Additionally, we perform a multiple linear regression model to understand if sex was associated with alterations in ASMI. As a result, age, sex, and BMI influence muscle mass in older adults: according to Table 2, ASMI decreases with age, male older adults have greater muscle mass than female older adults, and higher BMI is associated with an increase in ASMI.

These findings suggest that, within women, sarcopenia severity is primarily characterized by reductions in strength and functional performance rather than differences in muscle mass.

### 3.2. Differences in Muscle Strength Determined by Handgrip Strength Are Associated with Age and Sex

**Figure 2:**
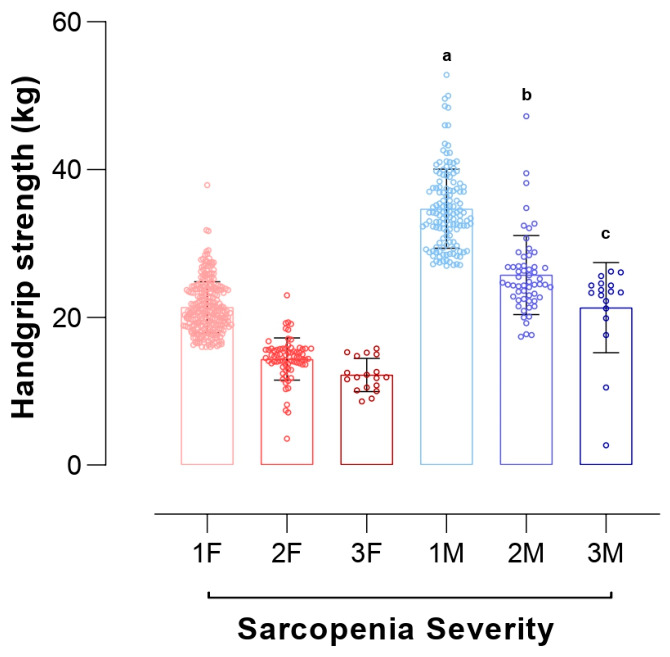
Muscle strength (kg) is higher in men than in women across all sarcopenia severity categories. The values for handgrip strength (kg) in each group are expressed as mean ± S.D. For females (red): 1F corresponds to mild sarcopenia (21.36 ± 3.46 kg, a); 2F corresponds to moderate sarcopenia (14.36 ± 2.84 kg, b); and 3F corresponds to severe sarcopenia (12.20 ± 2.23 kg, c). For males (blue): 1M corresponds to mild sarcopenia (34.71 ± 5.37 kg); 2M corresponds to moderate sarcopenia (25.74 ± 5.34 kg); and 3M corresponds to severe sarcopenia (21.30 ± 6.10 kg). Lowercase letters (a, b, c) indicate significant differences according to *a:* 1F vs. 1M; *b:* 2F vs. 2M; and *c:* 3F vs. 3M (*p*-values < 0.001).

Our results from Figure 2 demonstrate that men had significantly higher handgrip strength than women across all categories of sarcopenia severity (*p*-value < 0.001). Additionally, handgrip strength showed a progressive reduction with increasing sarcopenia severity in both sexes, while remaining substantially higher in men compared with women across all severity strata.

Moreover, we analyze whether other variables may be contributing to this effect. The results from Table 3 showed that age and sex are again factors associated with handgrip strength. For every year of age, handgrip strength decreases by 0.307 kg. Meanwhile, being male is associated with 12.41 more strength units than females (Table 3).

### 3.3. Physical Performance Determined by SPPB Is Associated with Both Age and BMI in Older Adults

**Figure 3:**
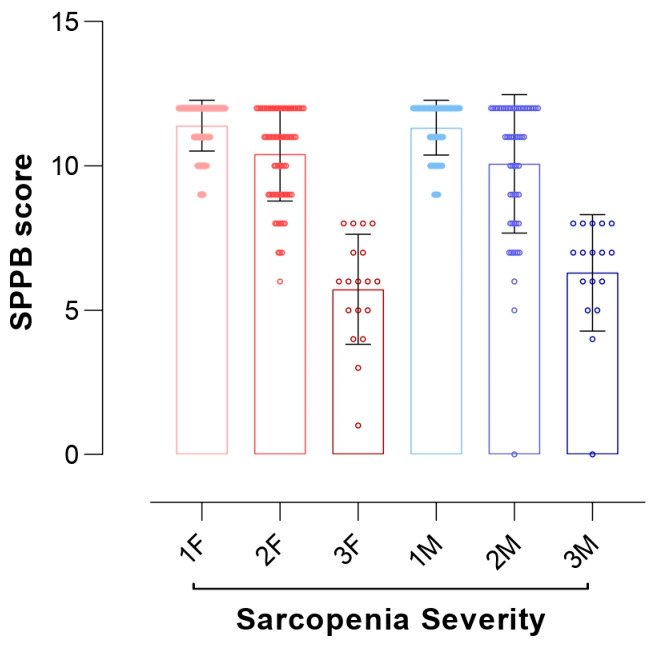
There are no significant differences in physical performance across sexes. This plot shows the physical performance, as evaluated by SPPB scores, across genders and severities of sarcopenia. The results demonstrate that, despite declines in physical performance in moderate and severe sarcopenia among both males and females, there are no significant sex differences. SPPB scores in each group are expressed as mean ± S.D. For females (red): 1F corresponds to mild sarcopenia (11.39 ± 0.88); 2F corresponds to moderate sarcopenia (10.40 ± 1.62); and 3F corresponds to severe sarcopenia (5.72 ± 1.90). For males (blue): 1M corresponds to mild sarcopenia (11.32 ± 0.95); 2M corresponds to moderate sarcopenia (10.07 ± 2.40); and 3M corresponds to severe sarcopenia (6.29 ± 2.02). No statistically significant differences in SPPB scores between men and women across any sarcopenia severity category (1F vs. 1M: *p*-value = 0.653; 2F vs. 2M: moderate: *p*-value = 0.872; and 3F vs. 3M: *p*-value = 0.252).

According to the results in Figure 3, SPPB is similar across genders when comparing sarcopenia severity groups.

Moreover, the multiple linear regression model (Table 4) showed negative associations of age and BMI with the SPPB score. This finding suggests that older age and higher BMI are associated with lower SPPB scores, indicating poorer functional performance. Furthermore, when controlling for other variables, there are no differences between sexes.

These findings indicate that functional performance, as measured by SPPB, decreases with increasing sarcopenia severity but does not differ significantly by sex within each severity stratum, suggesting that functional performance deficits may manifest similarly in both men and women despite differences in muscle mass and strength.

### 3.4. Age, but Not Sex, Leads to the “Moderate-to-Severe Sarcopenia” in Older Adults

Given the established clinical relevance of moderate and severe sarcopenia, these categories were combined into a single outcome (“moderate-to-severe sarcopenia”) for inferential analyses. In the binary logistic regression model, male sex was independently associated with higher odds of moderate-to-severe sarcopenia. Although the unadjusted regression model shows a statistically significant association between male and moderate-to-severe sarcopenia (OR 1.55; 95% CI 1.07–2.24; *p* = 0.021) (Supplementary Materials Table S1), when we adjust the binomial model by age, sex, BMI, and multimorbidity, the results indicate that only age is significantly associated with moderate-to-severe sarcopenia (Table 5).

### 3.5. Age, but Not Sex, Conditions the Severity of Sarcopenia in Older Adults

To preserve the ordinal structure of EWGSOP2 severity and to evaluate whether sex influences the severity of sarcopenia, we fitted a multinomial logistic regression model with the three uncollapsed severity categories. As a result, the OR of the unadjusted model (Supplementary Materials Table S2) indicated that men exhibited increased probabilities of moderate sarcopenia (OR = 1.49; 95% CI 0.994–2.23; *p*-value: 0.05). However, when the multinomial regression model was adjusted for age, sex, BMI, and multimorbidity, the data indicated that age significantly influences the likelihood of presenting with moderate or severe sarcopenia (Table 6).

Finally, an ordinal logistic regression model was fitted with the categories of sarcopenia severity as the response variable, indicating that for each additional year of age, the likelihood of developing a more severe form of the condition increases (Table 7).

## 4. Discussion

Sarcopenia has been suggested as a fundamental age-related condition that results in adverse outcomes since sarcopenia reduces the quality of life and is closely linked to chronic diseases and, in extreme cases, to mortality [21]. Currently, sarcopenia has a wide range of prevalence in adults over 60 years old. Such effects may be related to risk factors including sex, BMI, physical inactivity, malnutrition, extreme sleep duration, and osteoarthritis [22,23]. In fact, it has been reported that male individuals typically experience faster absolute muscle mass loss and a higher prevalence rate in early old age, whereas women face more severe functional consequences, a stronger link to cognitive decline, and a higher mortality risk [24]. In contrast, in Mexican-American populations, it has been suggested that older women have a higher prevalence of sarcopenia than men, but men exhibit a faster decline in muscle mass as compared to other populations. This effect may be due to socioeconomic factors, since according to Espinel-Bermudez et al., older adults with sarcopenia are distributed in rural zones highly marginalized and have poor accessibility to foods with high nutritional value [25].

In this study, we examined sex differences in the severity of sarcopenia and its core physiological domains—muscle mass, strength, and physical performance—using the EWGSOP2 criteria in 556 older adults from the Koltin cohort. Consistent with previous reports [14], our results show that sarcopenia prevalence is higher in women than in men. Nevertheless, our statistical analyses demonstrate that sex is not associated with disease severity. This effect may be driven by the fact that the EWGSOP2 cutoff points are standardized using healthy young European adults as the normative reference [2]. Hence, applying EWGSOP2 criteria blindly to Mexican older adults introduces a systematic sex bias; it frequently underestimates the severity of muscle mass loss in women (due to short stature and obesity masking) while potentially misclassifying functional strength boundaries in men. This underscores the critical need for population-specific, validated cutoffs that reflect the true biological and anthropometric realities of aging in Mexico.

Additionally, since neither the EWGSOP2 nor the AWGS cutoff values were originally derived from Mexican reference populations, prevalence estimates vary substantially depending on the consensus guidelines applied [26]; consequently, our findings should be interpreted within the specific context of this study population. While our observations align with previous literature reporting a high prevalence of moderate sarcopenia among older adults in Mexico City under the original EWGSOP framework [20], notable discrepancies emerge. Using the updated EWGSOP2 criteria, which refine the threshold values for grip strength and muscle mass [27], this study observed a lower prevalence of diagnosed sarcopenia among male participants than would be estimated with the older EWGSOP criteria [27,28].

On the other hand, our findings show that men exhibited higher muscle mass (ASMI) and handgrip strength (DINA) than women. Moreover, these results are consistent with those from our adjusted multiple linear regression model, which shows that ASMI was influenced by age, sex, and BMI, whereas handgrip strength was associated only with age and sex. The association between males and higher ASMI and handgrip strength was expected and consistent with age-related physiological profiles. Men typically maintain larger muscle mass and higher maximal strength throughout life, driven by androgen exposure, greater muscle fiber cross-sectional area, and higher absolute loading during daily activities [29]. It has been reported that men have greater grip strength than women, despite the decline in strength that begins in middle age [30]. Additionally, these results are consistent with previous reports indicating that men have greater muscle mass than women due to the gradual decline in hormones such as testosterone and insulin-like growth factor-1 (IGF-1), as reported by Guligowska et al. [31], suggesting that molecular mechanisms involved in sarcopenia may be different across males and females and deserve to be elucidated in further studies.

Our results concur with those of Yang et al. [32], indicating a positive association between BMI and ASMI. Interestingly, in individuals with high BMI, there are issues with classifying sarcopenia using ASMI due to increased adipose tissue [33,34]. However, our results show that participants’ BMI is within the normal range.

In contrast to ASMI and handgrip strength, the results from physical performance analysis (SPPB) show no significant differences between the sexes, a finding supported by the adjusted multiple linear regression model, which indicates that both age and BMI may influence SPPB. In this sense, it has been suggested that adults with high BMI and low muscle mass experience a synergistic loss of physical performance, as excess fat imposes additional mechanical stress on fragile joints and impairs dynamic balance. Also, the absence of sex differences in SPPB underscores the importance of integrating performance measures into routine assessment, as they may capture deficits not evident from muscle mass or strength alone. In this sense, two-stage screening methods have been proposed for sarcopenia, as defined by EWGSOP2, in older adults. These methods involve using standardized questionnaires, such as the SARC-F, followed by handgrip strength and bioelectrical impedance analysis (BIA) measurements. The latter includes the selective assessment of ASMI only in participants with positive screening results [35]. The aim of these methods is to efficiently and scalably identify sarcopenia, thereby facilitating the implementation of prevention strategies.

Our results from the SPPB are contrary to those previously reported in the literature, which mention that women are prone to functional limitations and sarcopenic obesity [36]. We suggest that these differences may reflect an inherent bias in our data, as membership in a private health insurance plan may provide critical financial and medical pathways to preserve and improve physical performance, even among individuals with sarcopenia [37]. Our group may address this hypothesis in future longitudinal studies.

Moreover, we attempted to identify variables associated with an increased likelihood of presenting with moderate or severe sarcopenia. Thus, we performed binomial, multinomial, and ordinal logistic regressions. According to our unadjusted multinomial regression results, males have a higher OR for moderate sarcopenia than females; however, after adjusting for age, sex, BMI, and multimorbidity, our results indicate that only age influences the likelihood of presenting with either moderate or severe sarcopenia. Similarly, the results from the adjusted ordinal regression model indicate that only age is significantly associated with an increased likelihood of developing severe sarcopenia, suggesting that, in our population, the severity of sarcopenia depends significantly on age rather than sex. These results are consistent with the literature, as aging is considered the primary cause of sarcopenia [38]. Aging not only drives sarcopenia but also contributes to broader systemic deterioration, disrupts metabolic balance, reduces type II fiber size and number, and alters the replacement and repair of damaged muscle fibers; in addition, hallmarks of aging, such as mitochondrial dysfunction and chronic inflammation, are associated with sarcopenia [21].

Although our study is an innovative piece that provides additional insight into the factors contributing to the wide range in the prevalence of sarcopenia among older adults, it has some limitations. For instance, the study does not include a control group without sarcopenia, thereby limiting the ability to draw conclusions about the progression of this condition relative to healthy aging trajectories and age-related functional decline. Our interpretation of the results is limited to a Mexican population of beneficiaries of private health insurance, and it would be interesting to validate these results in other foreign populations with similar characteristics. Another limitation of the study regards the cross-sectional design, since it does not allow inference about the progression or trajectories of sarcopenia over time. Finally, unmeasured confounding factors, including comorbidities, physical activity, hormonal status, and nutritional intake, may also influence age-related differences in severity.

## 5. Conclusions

Collectively, this study demonstrates that while older Mexican men within a private healthcare cohort maintain greater muscle mass and grip strength than women, physical performance metrics show no significant sex-based differences. Furthermore, after fully adjusting for covariates, advanced age—rather than sex—emerges as the primary driver of overall sarcopenia severity. These findings suggest that, to effectively mitigate severe muscle wasting, clinical interventions should prioritize older age groups, irrespective of sex, and place greater emphasis on comprehensive functional mobility assessments alongside traditional measures of muscle mass.

## Figures and Tables

**Table 1:** Characteristics of the study population.

Variable	Data
**Age** y.o. (mean ± SD)	72.27 ± 6.35
Mild sarcopenia age	70.97 ± 5.91
Moderate sarcopenia age	74.70 ± 6.23
Severe sarcopenia	77.97 ± 6.02
**Sex**	
Female *n* (%)	346 (62.23)
**Sarcopenia severity**	
Mild sarcopenia (Global) *n* (%)	392 (70.50)
**By Sex**	
Female	256 (65.30)
Male	136 (34.69)
Moderate sarcopenia *n* (%)	129 (23.20)
**By Sex**	
Female	72 (55.81)
Male	57 (40.31)
Severe sarcopenia *n* (%)	35 (6.29)
**By Sex**	
Female	18 (51.42)
Male	17 (48.57)
**BMI (kg/m^2^)** (mean ± SD)	24.43 ± 2.80
Mild sarcopenia	24.36 ± 2.64
Moderate sarcopenia	24.55 ± 3.12
Severe sarcopenia	24.89 ± 3.37
**ASMI (kg/m^2^) by Sarcopenia Severity** (mean ± SD)	
Mild sarcopenia	5.47 ± 0.80
Moderate sarcopenia	5.53 ± 0.86
Severe sarcopenia	5.42 ± 0.82
**Handgrip strength (kg) by Sarcopenia Severity** (mean ± SD)	
Mild sarcopenia	25.99 ± 7.62
Moderate sarcopenia	19.38 ± 7.00
Severe sarcopenia	16.62 ± 6.42
**SPPB score by Sarcopenia Severity** (mean ± SD)	
Mild sarcopenia	11.36 ± 0.90
Moderate sarcopenia	10.25 ± 1.99
Severe sarcopenia	6.00 ± 1.95
**Multimorbidity** *n* (%)	96 (17.26)
Mild sarcopenia	60 (15.30)
Moderate sarcopenia	29 (22.48)
Severe sarcopenia	7 (20)
**Total of participants (*n*)**	556

**Table 2:** Adjusted model of the multiple linear regression including ASMI as the dependent variable.

Term	B	95% CI	β	*p*-Value
Age (y.o)	−0.005	−0.010, −0.0005	−0.043	0.028
Male vs. Female	1.385	1.319, 1.451	1.759	6.32 × 10^−170^
BMI (kg/m^2^)	0.070	0.059, 0.082	0.070	7.90 × 10^−31^
Multimorbidity	−0.031	−0.114, 0.052	0.024	0.462
Intercept	3.637	3.197,4.077		9.89 × 10^−49^

**Table 3:** Adjusted model of the multiple linear regression including handgrip strength as the dependent variable.

Term	B	95% CI	β	*p*-Value
Age (y.o)	−0.307	−0.380, −0.234	−0.240	9.83 × 10^−16^
Male vs. Female	12.411	11.448, 13.373	1.447	1.88 × 10^−94^
BMI (kg/m^2^)	−0.016	−0.181, 0.148	0.070	0.842
Multimorbidity	0.086	−1.123, 1.297	0.013	0.888
Intercept	41.805	35.409, 48.200		3.39 × 10^−33^

**Table 4:** Adjusted model of multiple linear regression including SPPB as the dependent variable.

Term	B	95% CI	β	*p*-Value
Age (y.o)	−0.091	−0.114, −0.068	−0.311	6.14 × 10^−14^
Male vs. Female	−0.047	−0.354, 0.025	−0.168	0.7624
BMI (kg/m^2^)	−0.078	−0.131, −0.025	−0.118	0.003
Multimorbidity	−0.359	−0.746, 0.026	−0.236	0.067
Intercept	19.378	17.337, 21.420		1.67 × 10^−60^

**Table 5:** Adjusted binomial regression model. OR: odds ratio; CI: confidence interval.

Term	Estimate (OR)	95% CI	*p*-Value
Male vs. Female	1.26	0.843–1.87	0.26
Age (y.o)	1.13	1.09–1.17	6.72 × 10^−12^
BMI (kg/m^2^)	1.01	0.943–1.08	0.761
Multimorbidity	1.39	0.846–2.25	0.189
Intercept	0.00003	0.000001–0.0006	3.63 × 10^−11^

**Table 6:** Adjusted multinomial regression model. OR: odds ratio; CI: confidence interval.

Level	Term	Estimate (OR)	95% CI	*p*-Value
Moderate vs. Mild	Male vs. Female	1.25	0.815–1.91	0.309
Moderate vs. Mild	Age (y.o.)	1.11	1.07–1.15	5.38 × 10^−8^
Moderate vs. Mild	BMI (kg/m^2^)	1	0.933–1.08	0.911
Moderate vs. Mild	Multimorbidity	1.45	0.865–2.43	0.159
Moderate vs. Mild	Intercept	0.0001	6.07 × 10^−6^–0.003	6.39 × 10^−8^
Severe vs. Mild	Male vs. Female	1.34	0.64–2.79	0.44
Severe vs. Mild	Age (y.o.)	1.24	1.15–1.33	5.62 × 10^−9^
Severe vs. Mild	BMI (kg/m^2^)	1.04	0.915–1.18	0.54
Severe vs. Mild	Multimorbidity	1.15	0.461–2.88	0.762
Severe vs. Mild	Intercept	2.93 ×10^−9^	4.77 × 10^−12^–1.8 × 10^−6^	1.99 × 10^−9^

**Table 7:** Adjusted model of the ordinal logistic regression model. OR: odds ratio; CI: confidence interval.

Term	Estimate (OR)	95% CI	*p*-Value
Age (y.o)	1.137	1.099–1.177	3.198 × 10^−13^
Male vs. Female	1.249	0.847–1.842	0.263
BMI (kg/m^2^)	1.008	0.942–1.078	0.821
Multimorbidity	1.348	0.841–2.159	0.214
Intercept Mild|Moderate	40,926.15	2096.99–798,738.99	2.47 × 10^−12^
Intercept Moderate|Severe	302,053.89	14,519–6,283,939.96	3.68 × 10^−16^

## Data Availability

Data are available upon reasonable request due to privacy restrictions.

## Supplementary Materials

The following supporting information can be downloaded at https://www.mdpi.com/article/10.3390/geriatrics11040077/s1; Table S1: Unadjusted binomial regression model. OR: odds ratio; CI: confidence interval; Table S2: Unadjusted model of multinomial regression. OR: odds ratio; CI: confidence interval.

## Abbreviations

The following abbreviations are used in this manuscript:

EWGSOP2	European Working Group on Sarcopenia in Older People 2
DEXA	Dual-Energy X-ray Absorptiometry
SPPB	Short Physical Performance Battery
AWGS	Asian Working Group for Sarcopenia
ASM	Appendicular skeletal muscle mass
ASMI	Appendicular skeletal muscle mass index
DINA	Handgrip strength
OR	Odds ratio
CI	Confidence intervals
F	Female
M	Male
