# Supplementary material for: Age, Not Sex, Drives Sarcopenia Severity in Mexican Older Adults with a Health Insurance Plan
Source: Geriatrics (Basel). 2026 Jul 1;11(4):77. doi: 10.3390/geriatrics11040077 (PMC13398084; doi:10.3390/geriatrics11040077)
Supplement: Supplementary file 1 [file geriatrics-11-00077-s001.zip › geriatrics-4303879-supplementary.pdf]

Supplementary Materials

Table S1. Unadjusted binomial regression model. OR: odds ratios; CI: Confidence intervals.

Term	Estimate (OR)	95% CI	p-value
Male vs Female	1.55	1.07-2.24	0.021**
Intercept	0.35	0.27-0.44	1.457E-17

Table S2. Unadjusted model of multinomial regression. OR: odds ratios; CI: Confidence intervals.

Level	Term	Estimate (OR)	95% CI	p-value
Moderate vs Mild	Intercept	0.281	0.217-0.365	1.921E-21
Moderate vs Mild	Male vs Female	1.490	0.994-2.23	0.05 *
Severe vs Mild	Intercept	0.070	0.044-0.113	1.33E-27
Severe vs Mild	Male vs Female	1.78	0.887-3.56	0.105
